# Supplementary material for: Single-cell transcriptomics reveals the molecular basis of human iPS cell differentiation into ectodermal ocular lineages
Source: Commun Biol. 2024 Nov 12;7:1495. doi: 10.1038/s42003-024-07130-4 (PMC11557866; doi:10.1038/s42003-024-07130-4)
Supplement: Supplementary file 1 — Supplementary Information [file 42003_2024_7130_MOESM1_ESM.pdf]

Supplementary Information for:

Single-Cell Transcriptomics Reveals the Molecular Basis of Human iPS Cell Differentiation into Ectodermal Ocular Lineages

Laura Howard,[1,2] Yuki Ishikawa,[3, 5] Tomohiko Katayama,[3, 5] Sung-Joon Park,[4] Matthew J. Hill,[2] Derek J. Blake,[2] Kohji Nishida,[5,6] Ryuhei Hayashi,[3, 5] Andrew J. Quantock[1]

[1] School of Optometry and Vision Sciences, Cardiff University, Cardiff, Wales, UK.

[2] Centre for Neuropsychiatric Genetics and Genomics, School of Medicine, Cardiff University, Cardiff, Wales, UK.

[3] Department of Stem Cells and Applied Medicine, Osaka University Graduate School of Medicine, Osaka, Japan.

[4] Institute of Medical Science, University of Tokyo, Tokyo, Japan.

[5] Department of Ophthalmology, Osaka University Graduate School of Medicine, Osaka, Japan.

[6] Institute for Open and Transdisciplinary Research Initiatives, Osaka University, Osaka, Japan.

Correspondence: Kohji Nishida, Ryuhei Hayashi

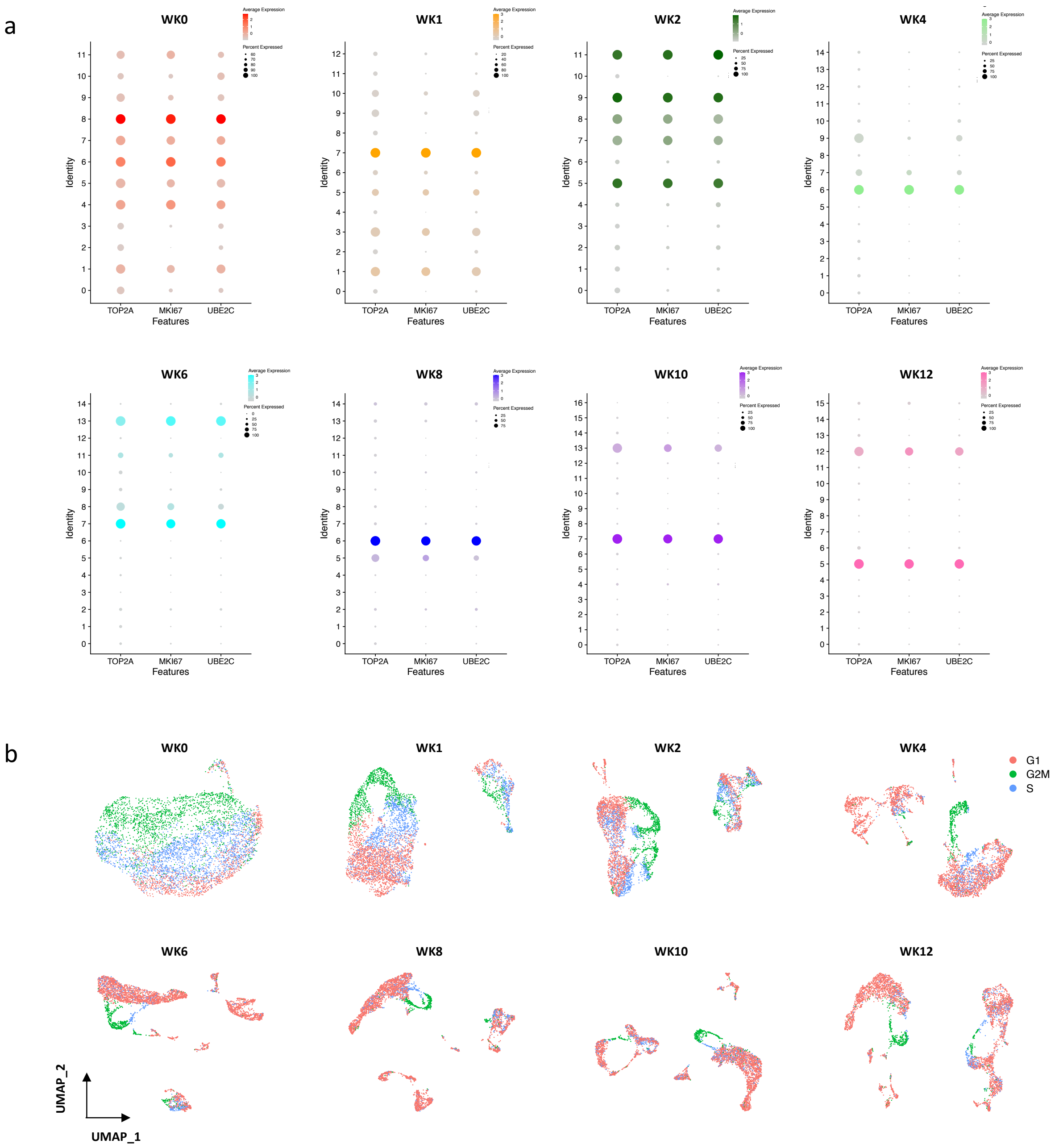

Supplementary Fig. 1: Cell cycle analysis during SEAM formation and development. (a) Dot plots showing cluster-specific expression of TOP2A, MKI67 and UBE2C at each developmental stage. (b) Feature plots showing proportion of cells in G1, G2/M and S phases of the cell cycle.

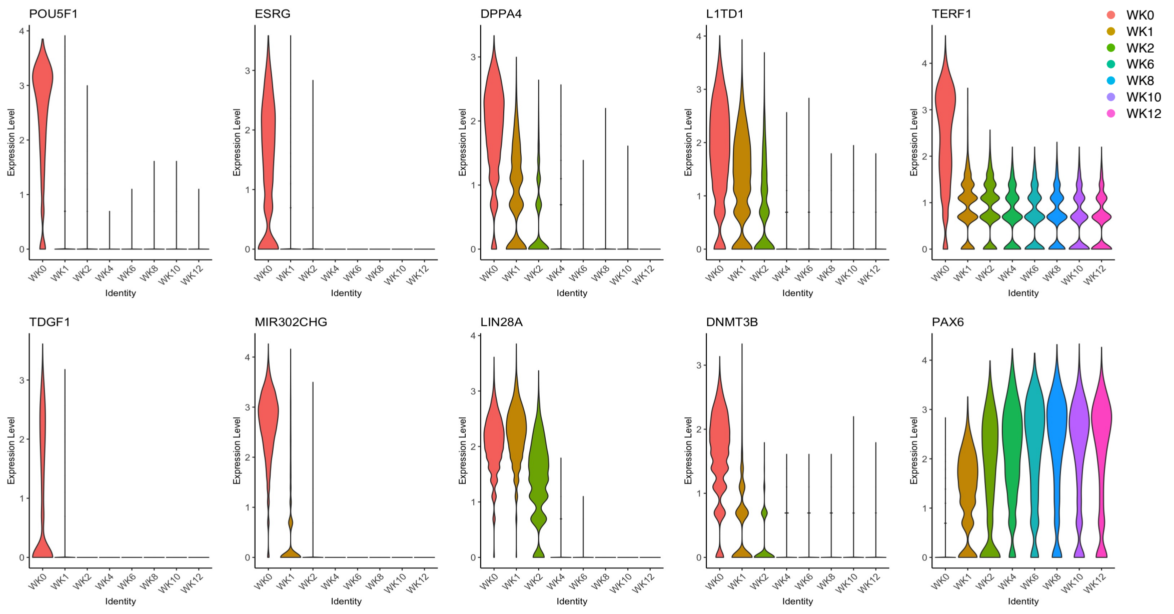

Supplementary Fig. 2: Progressive loss of pluripotency-associated genes over time. Violin plots showing expression levels of candidate pluripotency markers at each developmental stage. Reciprocal expression of PAX6 is also shown.

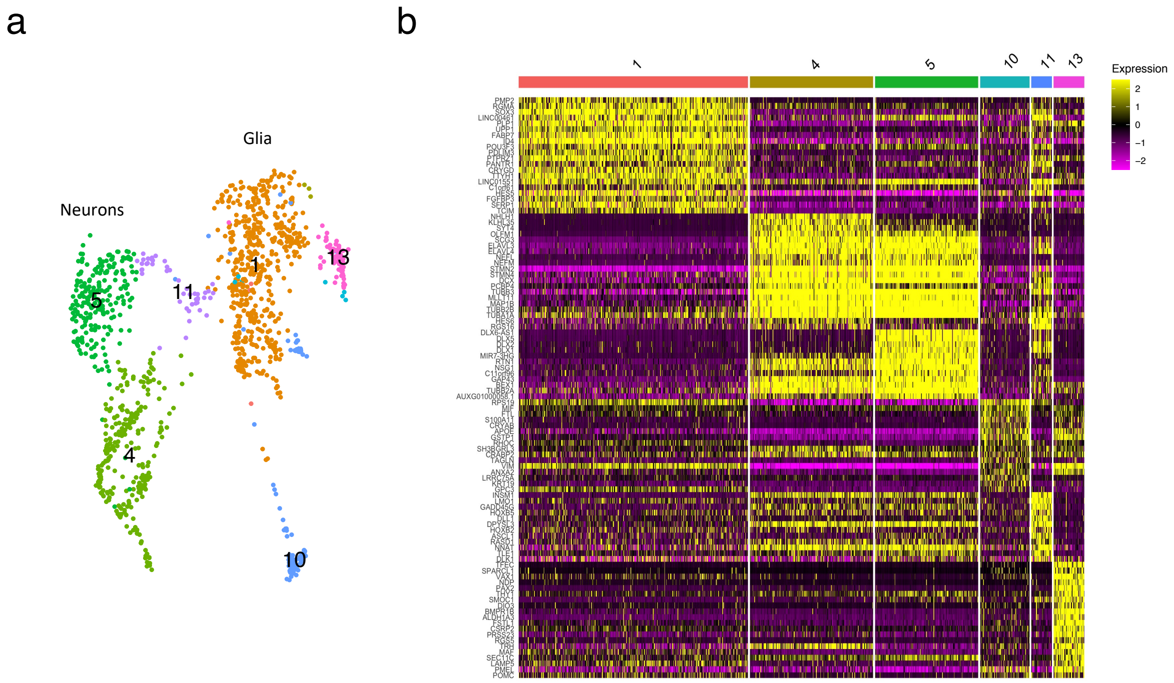

Supplementary Fig. 3: Neuronal and glial expression in WK4 SEAMs. (a) UMAP plots of neuronal and glial cell populations in WK4 SEAMs. (b) Heatmap showing the top markers expressed by cells in these clusters.

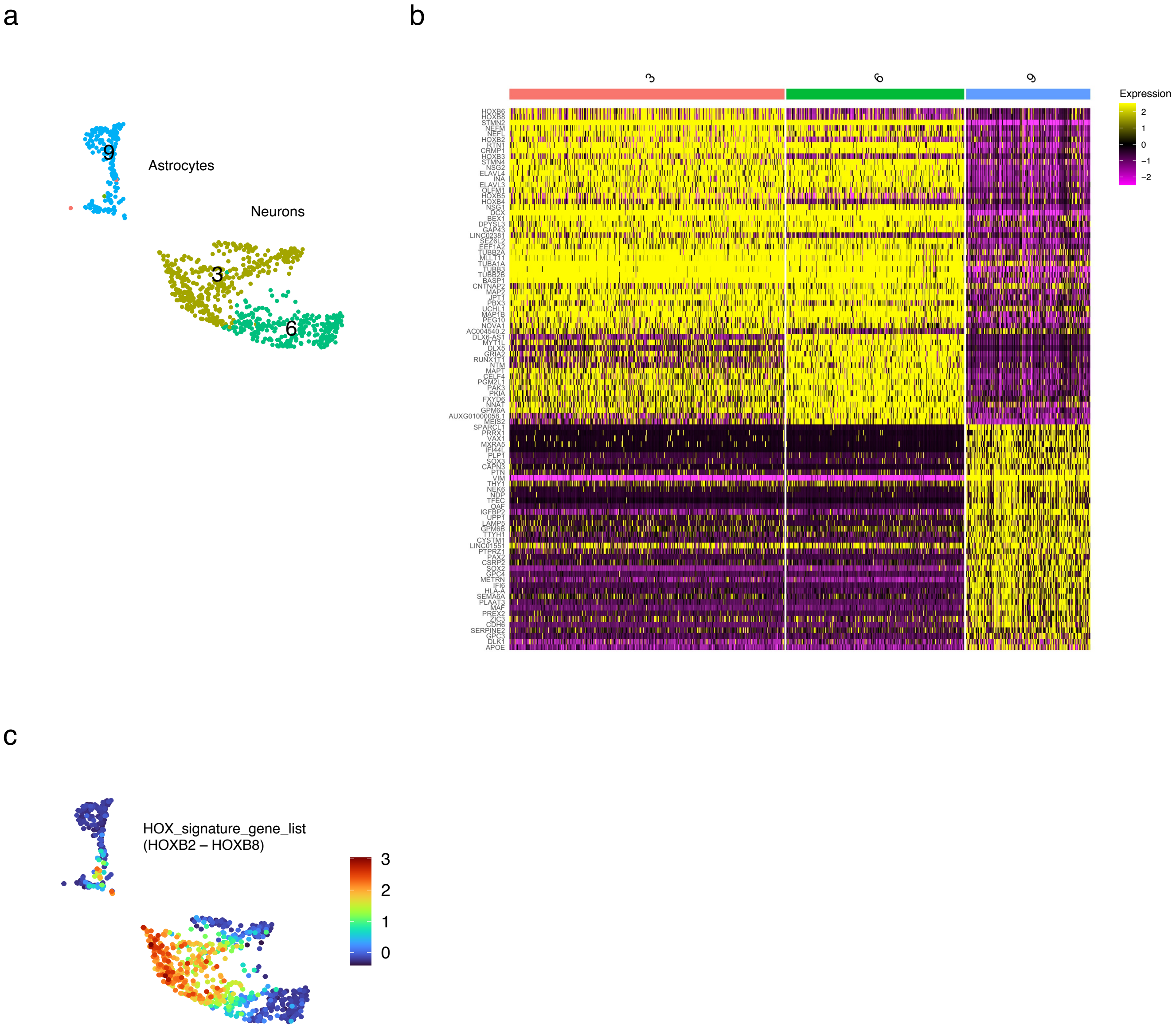

Supplementary Fig. 4: Cluster separation of neurons and astrocytes in WK6 SEAMs. (a) UMAP plots of neuron and astrocyte populations in WK6 SEAMs. (b) Heatmap showing the top markers expressed by cells in these clusters. Expression of the ocular markers PAX6, RAX, SIX6, VSX2 and SOX2 is also shown. (c) Feature plot showing module scoring density for HOX genes (HOXB2, HOXB3, HOXB4, HOXB5, HOXB6, HOXB7, HOXB8) in these cells.

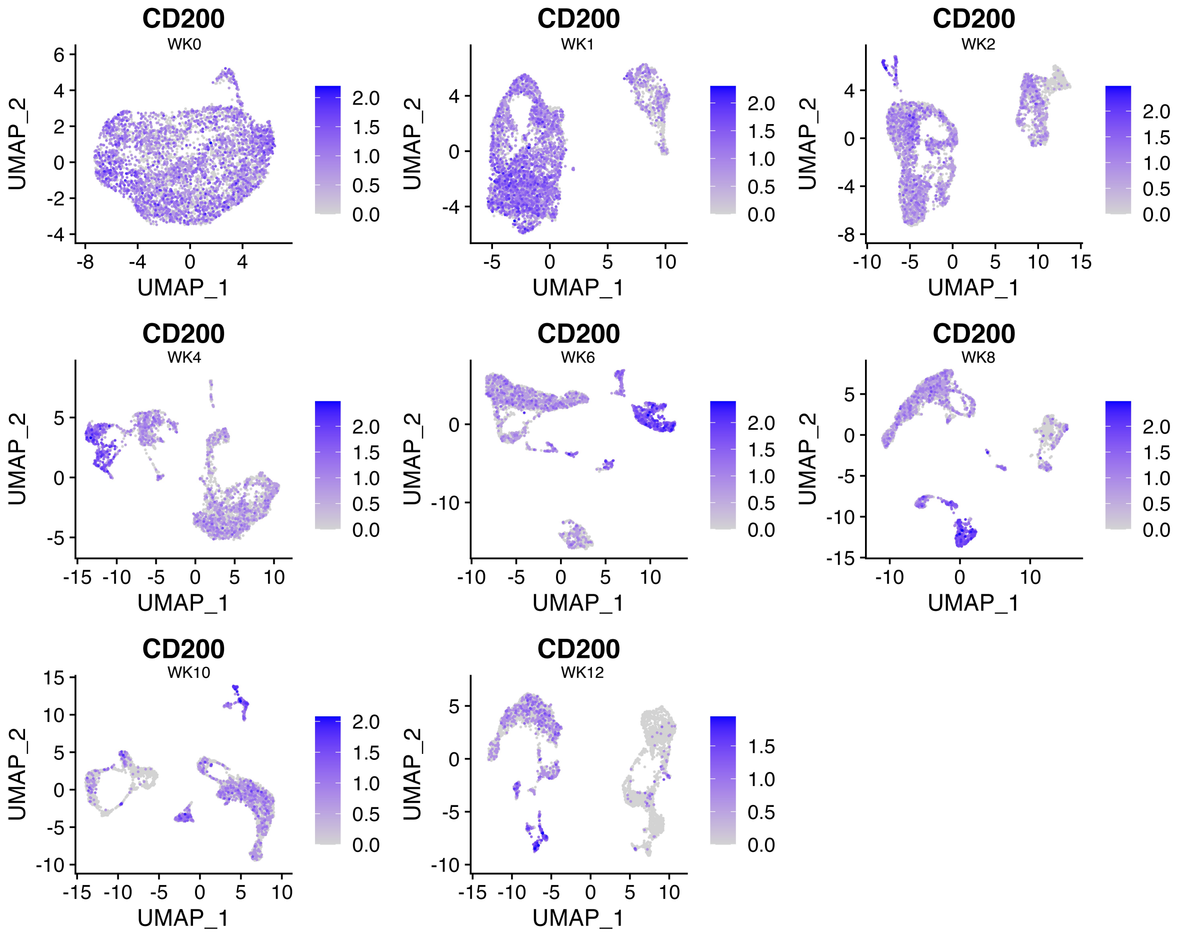

Supplementary Fig. 5: Expression of CD200 during SEAM development. Feature plots showing expression of CD200 from WK0 – WK12.

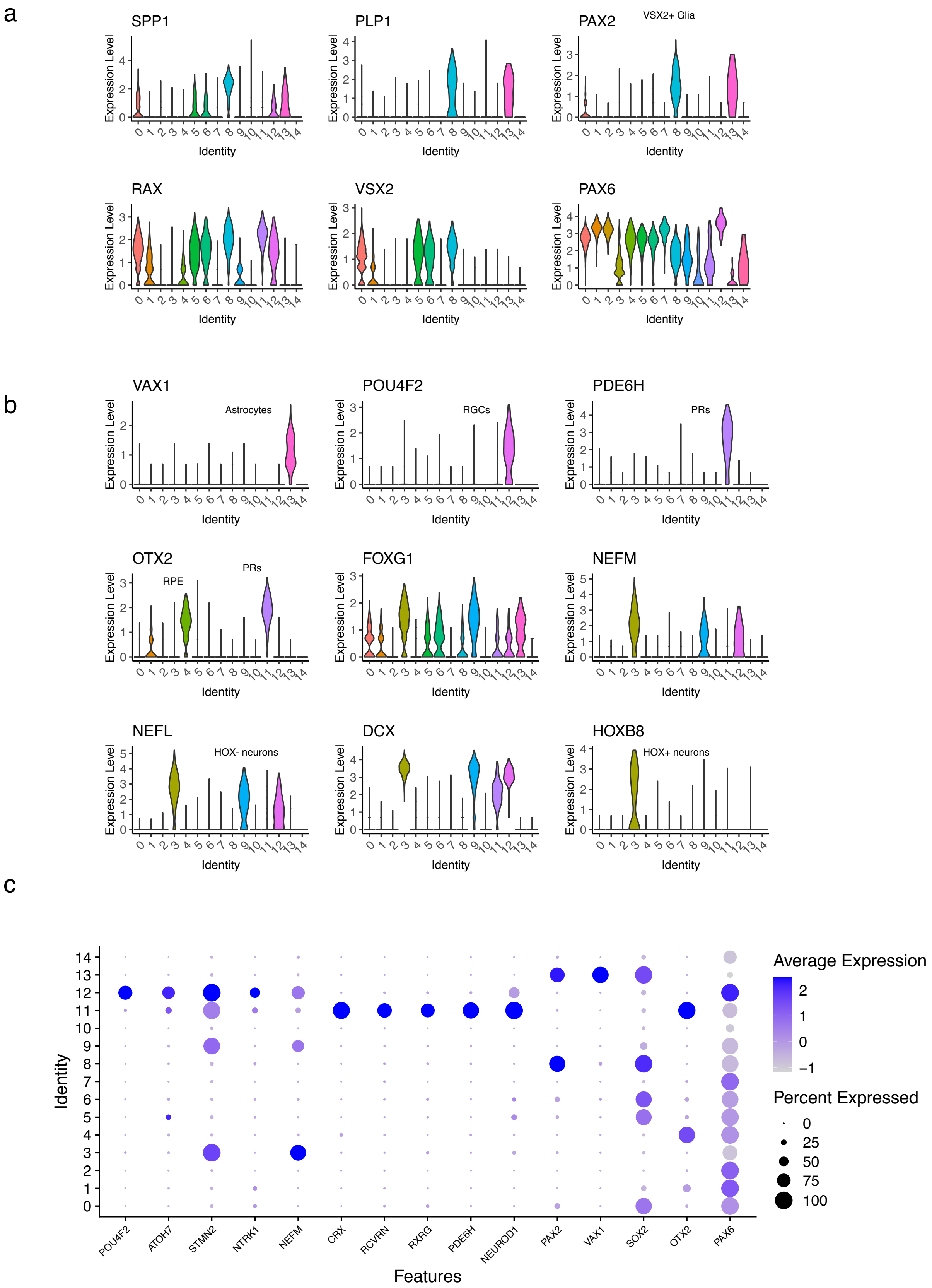

Supplementary Fig. 6: Cluster-specific expression of specialised ocular markers in WK8 SEAMs. (a) Violin plots showing co-expression of glial (PLP1, PAX2), RPC (VSX2) and ocular (RAX, PAX6) markers in cells in cluster 8, indicating a Müller glial-like identity. (b) Violin plots showing cell-specific expression of key ocular and neural markers. (c) Dot plot showing expression of RGC, photoreceptor and astrocyte markers in WK8 SEAMs.

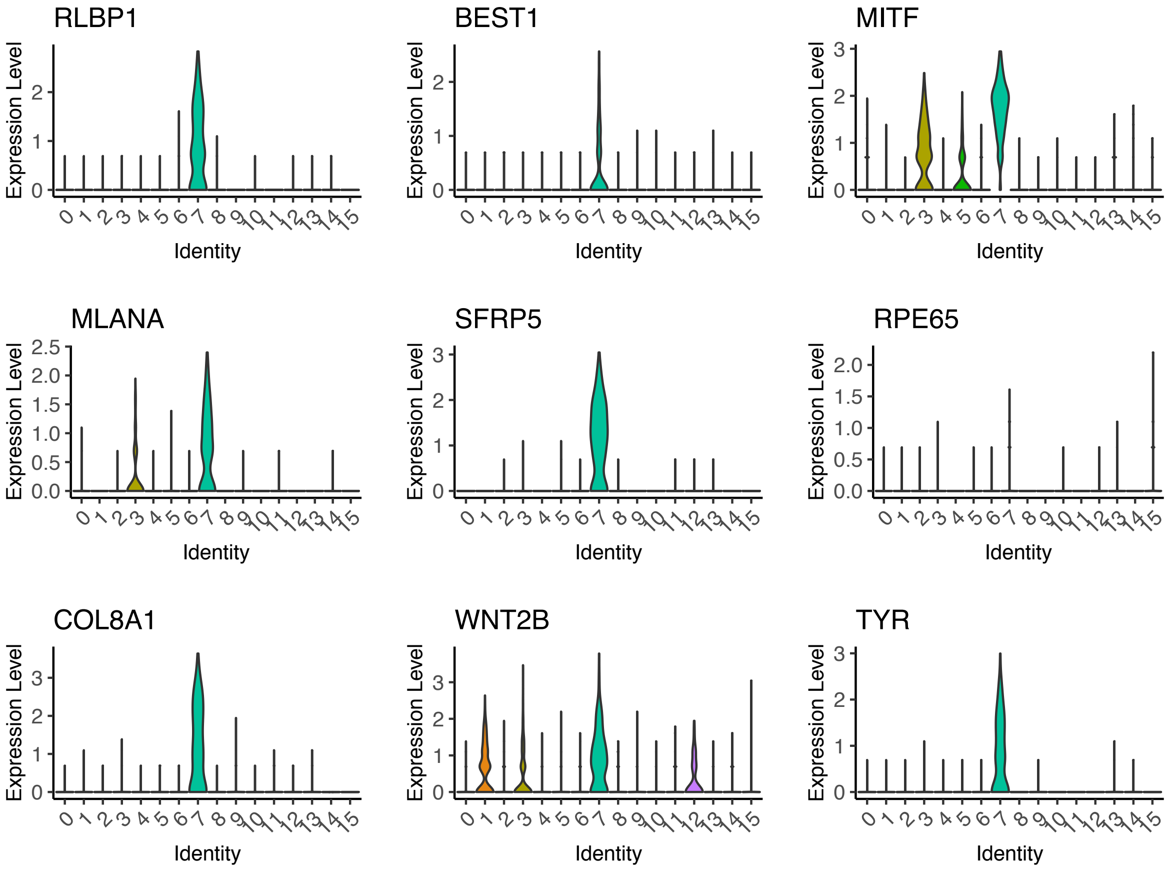

Supplementary Fig. 7: Cluster-specific expression of RPE markers in WK12 SEAMs. Violin plots showing co-expression of RPE markers in cluster 7.

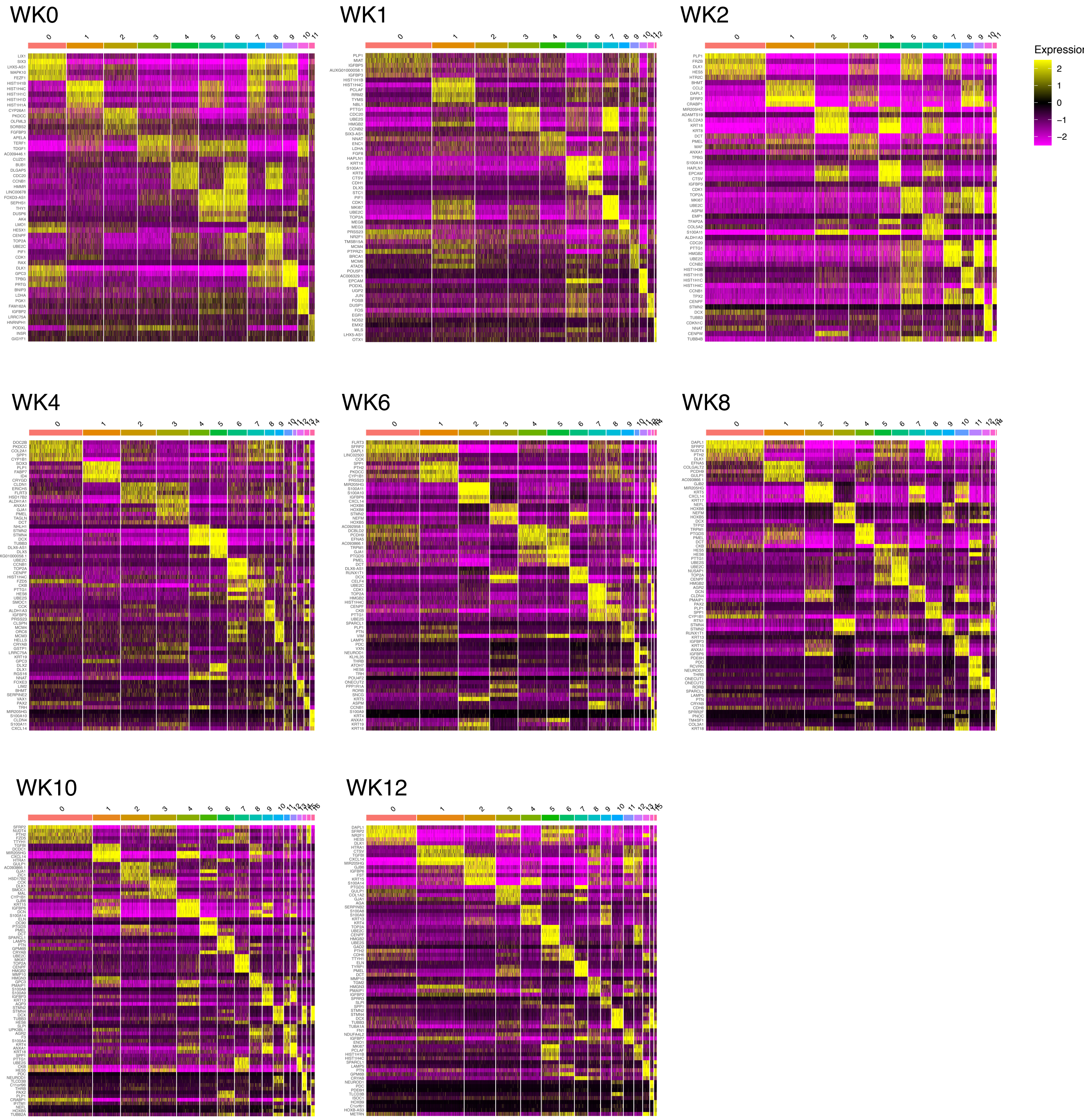

Supplementary Fig. 8: Heatmaps showing gene expression at all SEAM stages. Heatmaps showing the top_n = 5 genes returned by FindAllMarkers for each returned cluster at each developmental stage studied. Full gene lists are shown in Supplementary Data 1.

a

b

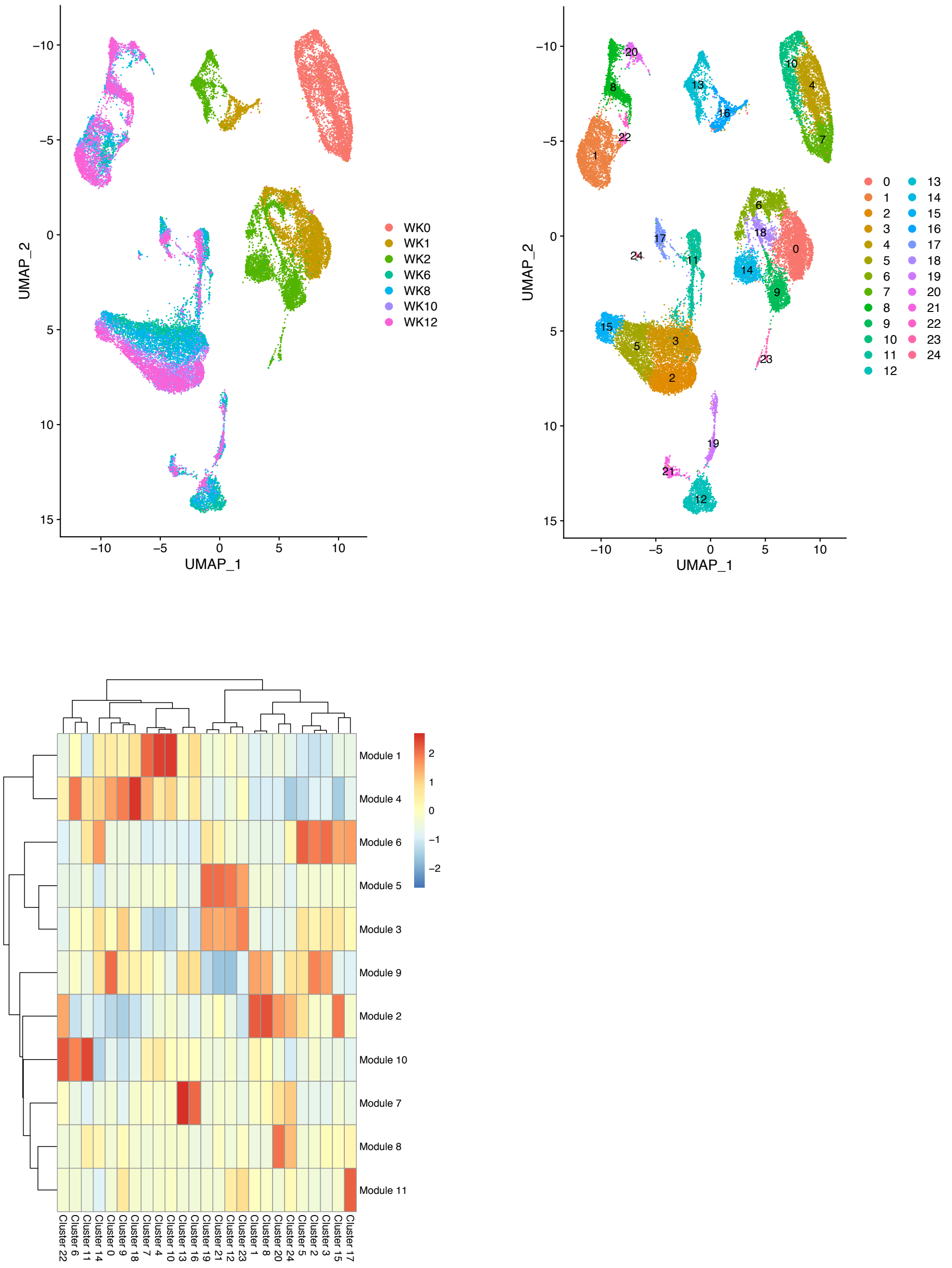

Supplementary Fig. 9: Integrative analysis of SEAM development. (a) UMAP plots of combined data, labelled according to sample identity and Seurat cluster. (b) Monocle3 analysis showing modules of co-regulated genes which change as a function of pseudotime. Full gene lists are shown in Supplementary Data 4.

a

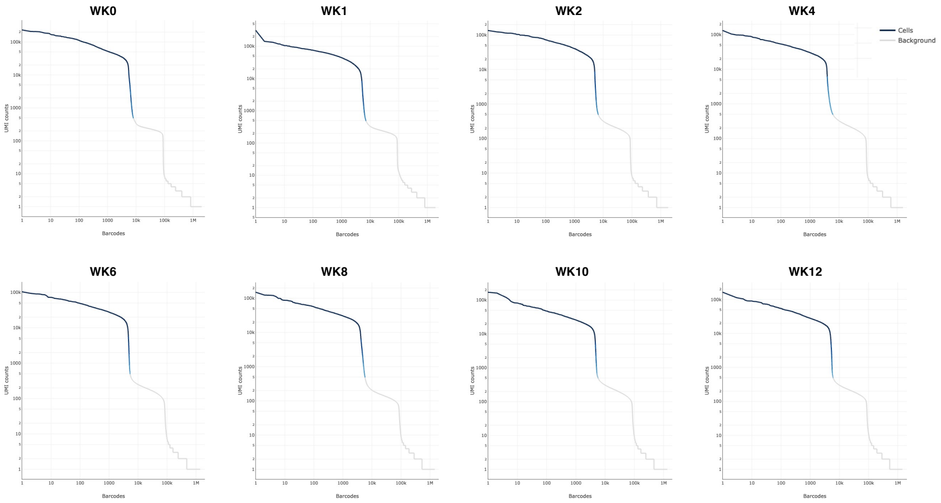

b

	WK0	WK1	WK2	WK4	WK6	WK8	WK10	WK12
**Estimated Number of Cells**	7144	6293	5965	4669	5029	5039	5046	5594
**Mean Reads per Cell**	93200	109249	130805	166938	135684	156389	146292	128893
**Median Genes per Cell**	6262	5513	5663	5178	4870	5041	4546	4428
**Valid Barcodes**	97.9%	97.8%	97.6%	97.4%	96.7%	96.9%	96.2%	96.2%
**Valid UMIs**	100.0%	100.0%	100.0%	100.0%	99.9%	99.9%	99.9%	99.9%
**Q30 Bases in Barcode**	95.8%	95.8%	96.0%	95.9%	95.8%	95.9%	95.9%	95.9%
**Q30 Bases in RNA Read**	92.0%	91.3%	92.0%	92.2%	91.4%	91.1%	91.5%	91.7%
**Q30 Bases in UMI**	95.2%	95.3%	95.4%	95.3%	95.3%	95.4%	95.5%	95.5%
**Reads Mapped to Genome**	96.8%	96.8%	96.1%	95.4%	95.1%	96.1%	94.1%	95.2%
**Reads Mapped Confidently to Genome**	95.0%	94.5%	93.5%	91.5%	92.3%	92.8%	89.9%	90.9%
**Fraction Reads in Cells**	91.7%	89.3%	91.1%	88.4%	91.7%	91.3%	90.8%	90.8%
**Number of Cells Passing QC in Seurat**	5153	4904	4836	3711	4188	3710	4260	4440

c

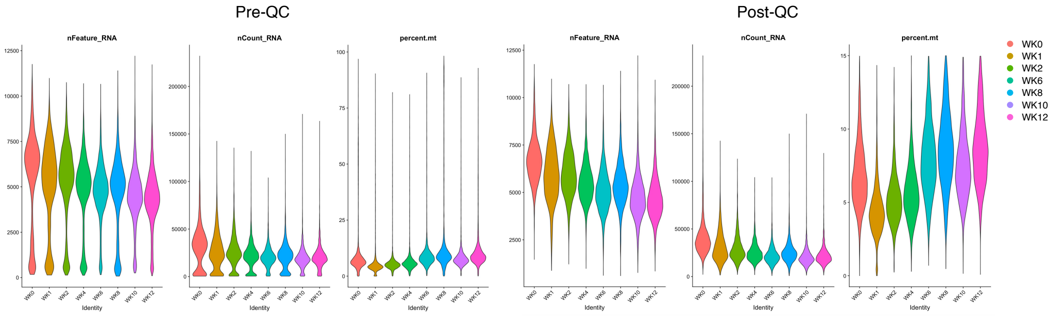

Supplementary Fig. 10: Quality control and selection of cells for analysis. (a) Barcode rank plots showing distribution of UMI counts in barcodes. (b) Sequencing metrics. (c) Violin plots showing QC plots pre- and post- QC processing.

Supplementary Table 1

Composition of culture medium for SEAM culture

DM (differentiation medium)

Reagent	Manufacturer	Cat. No.	Final concentration
G-MEM	Life Technologies	11710035	n/a
L-Glutamine 200mM (100X)	Life Technologies	25030081	2 mM
MEM Non-Essential Amino Acids Solution (100X)	Life Technologies	11140050	0.1 mM
Sodium pyruvate (100mM)	Life Technologies	11360070	1 mM
StemSureR 50mmol/L Monothioglycerol Solution (×100)	Fujifilm Wako	195-15791	55 μM
KnockOut™ Serum Replacement	Life Technologies	10828028	20%
PENICILLIN G POTASSIUM	Meiji Seika Pharma, Tokyo, Japan	876111	100 unit/mL
STREPTOMYCIN SULFATE	Meiji Seika Pharma	876161	0.1 mg/ml

CDM (corneal differentiation medium)

Reagent	Manufacturer	Cat. No.	Final concentration
DM (differentiation medium)	self-produced	n/a	50%
CnT-Prime (w/o; EGF and FGF)	CELLnTEC Advanced Cell Systems, Bern, Switzerland	CnT-PR-EF	50%
Recombinant Humman KGF	Fujifilm Wako	112-00813	20 ng/ml
CultureSure® Y-27632	Fujifilm Wako	030-24-026	10 μM
PENICILLIN G POTASSIUM	Meiji Seika Pharma	876111	100 unit/mL
STREPTOMYCIN SULFATE	Meiji Seika Pharma	876161	0.1 mg/ml

CEM (corneal epithelial maintenance medium)

Reagent	Manufacturer	Cat. No.	Final concentration
DMEM/F12	Life Technologies	11320033	n/a
Recombinant Humman KGF	Fujifilm Wako	11200813	20 ng/ml
CultureSure® Y-27632	Fujifilm Wako	030-24-026	10 μM
B-27™ Supplement (50X), serum free	Life Technologies	17504-044	2%
PENICILLIN G POTASSIUM	Meiji Seika Pharma	876111	100 unit/mL
STREPTOMYCIN SULFATE	Meiji Seika Pharma	876161	0.1 mg/ml
