# Supplementary material for: Single-cell transcriptomics reveals the molecular basis of human iPS cell differentiation into ectodermal ocular lineages
Source: Commun Biol. 2024 Nov 12;7:1495. doi: 10.1038/s42003-024-07130-4 (PMC11557866; doi:10.1038/s42003-024-07130-4)
Supplement: Supplementary file 2 — Description of Additional Supplementary Files [file 42003_2024_7130_MOESM2_ESM.pdf]

Description of Additional Supplementary Files

File name: Supplementary Data 1
Description: Cluster-specific differential expression analysis for each individual time point studied. Data were generated using the Seurat FindAllMarkers function.

File name: Supplementary Data 2
Description: Differential expression analysis for all identity classes (combined data). Data were generated using the Seurat PrepSCTFindMarkers followed by the FindAllMarkers function, and are shown annotated by both Seurat cluster and sample identity.

File name: Supplementary Data 3
Description: Monocle3 module groupings indicating co-regulated genes. Expression analysis was performed using graph_test() with neighbor_graph=”knn” and modules of co-regulated genes were extracted using find_gene_modules with resolution=1e-4.

File name: Supplementary Data 4
Description: Monocle3 module groupings indicating co-regulated genes which change as a function of pseudotime. Expression analysis was performed using graph_test() with neighbor_graph=”principal_graph” and modules of co-regulated genes were extracted using find_gene_modules with resolution=1e-4.
